# Invasive Fungal Rhinosinusitis in the Era of COVID-19: Is It an Emerging Association?

**DOI:** 10.7759/cureus.27222

**Published:** 2022-07-25

**Authors:** Ipek Chatzisouleiman, Stergios Lialiaris, Maria Zisoglou, Melina Katsilidou, Michail Katotomichelakis

**Affiliations:** 1 Otolaryngology - Head and Neck Surgery, Democritus University of Thrace-General University Hospital of Alexandroupolis, Alexandroupolis, GRC

**Keywords:** fungal, aspergillus, immunosufficiency, rhinosinusitis, invasive, covid-19

## Abstract

Invasive fungal rhinosinusitis (IFRS) typically affects immunocompromised patients. The coronavirus disease 2019 (COVID-19) pandemic may be associated with rare opportunistic fungal infections, probably as a result of immune dysregulation. The COVID-19 infection is characterized by low levels of CD4+T and CD8+T cells which could increase the risk of co-infections from *Mucor *or *Aspergillus *species. An invasive fungal infection should be suspected in patients who have recently recovered from COVID-19 pneumonia and present with acute destructive rhinosinusitis. There are few cases of IFRS reported in Europe during the pandemic of COVID-19. We describe the case of a 67-year-old patient with diabetes who received corticosteroids during the treatment for COVID-19 infection and was readmitted a few days later for radiologically and clinically suggested IFRS. *Aspergillus niger* was identified, and the patient received pharmacological and surgical treatment.

## Introduction

Yeast and bacteria are organisms that can be found in the normal flora of a human body. If the environment is suitable, the fungi can cause pathological conditions such as pneumonia and acute fungal rhinosinusitis. Fungal rhinosinusitis is categorized into two broad categories, namely, invasive and non-invasive, based on the potential to penetrate the surrounding epithelium. Acute invasive fungal rhinosinusitis (IFRS) is defined as “the presence of fungal hyphae within the sinonasal mucosa, submucosa, vasculature or bone in the setting of one month or less of sinusitis symptoms” [1]. The species that lead to this disease belong to the following two categories: *Zygomycetes *and *Aspergillus*. There is emerging evidence for the rise of this co-infection during the coronavirus disease 2019 (COVID-19) pandemic in an immunosuppressed environment. Here, we present the case of a diabetic patient with acute invasive fungal rhinosinusitis following a recent recovery from severe acute respiratory syndrome coronavirus 2 (SARS-CoV-2).

## Case presentation

**Figure 1:**
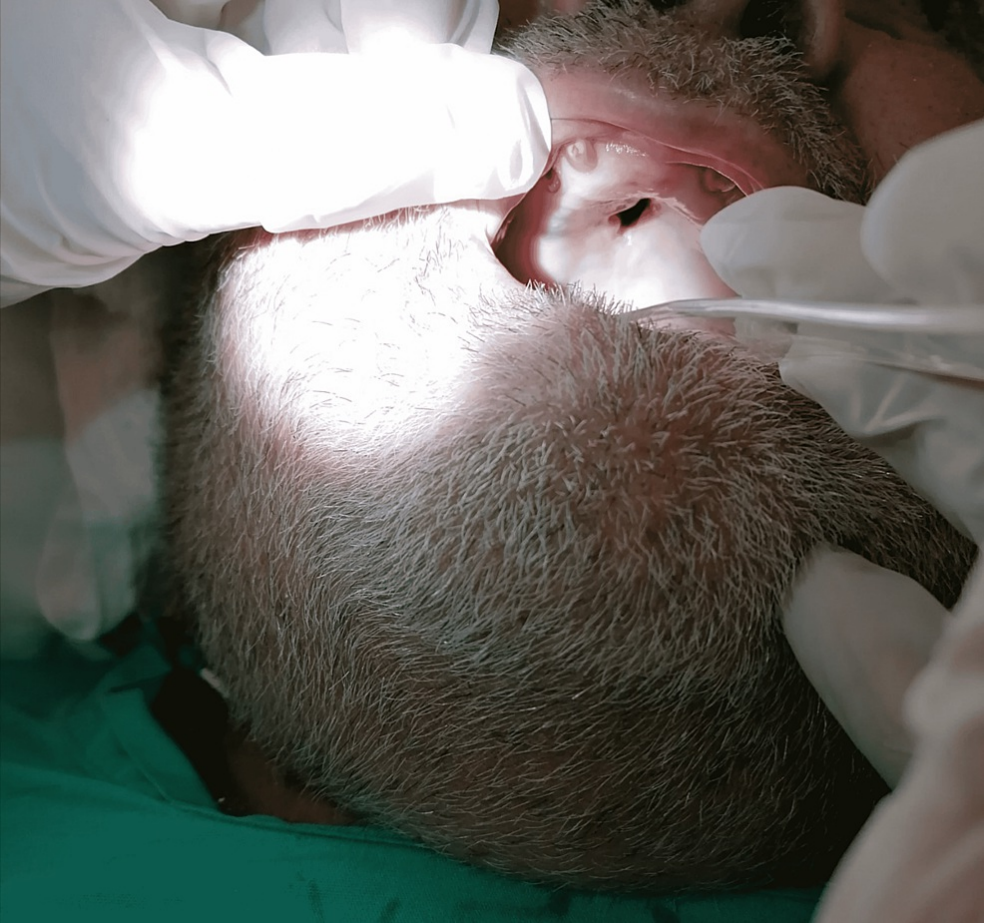
Necrotic part of the hard palate.

**Figure 2:**
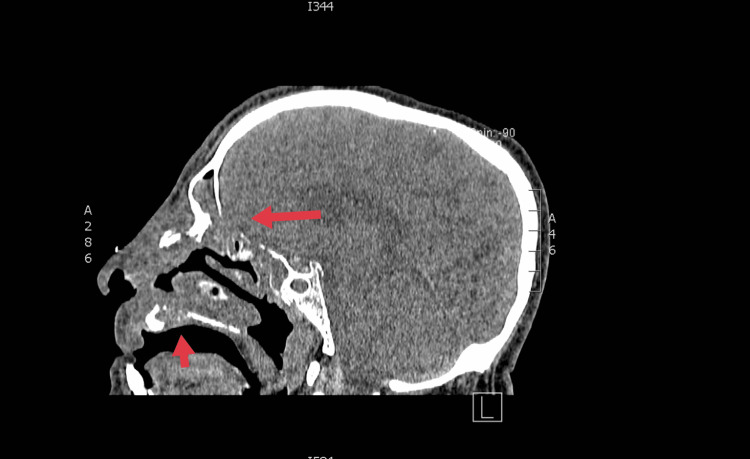
Sagittal plane: Cranial computed tomography scan. Arrows indicate the osteolytic regions of the hard palate and posterior table of the frontal sinus.

**Figure 3:**
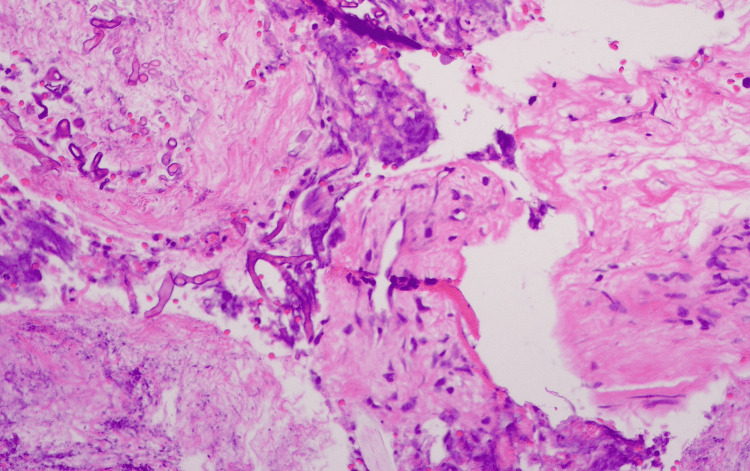
Histopathology results: Necrotic tissue and mycelia.

**Figure 4:**
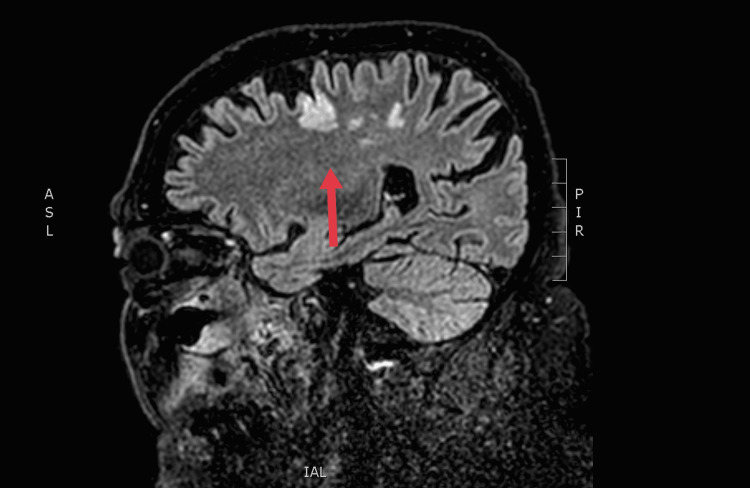
Brain magnetic resonance imaging of the patient: Fluid-attenuated inversion recovery after the first surgical intervention. The arrow shows the ischemic regions of the brain.

A 67-year-old male, with poorly controlled diabetes mellitus, renal, and cardiac failure, was referred to our department with clinically suggestive and radiologically proven pansinusitis. In the emergency department, clinical examination revealed a disoriented patient with facial asymmetry during speech and left arm weakness. He showed mild respiratory distress due to the difficulty with nasal breathing, right eye proptosis with periorbital swelling, and ophthalmoplegia. Nasal and mouth examination revealed purulent nasal discharge and a necrotic ulcer on the hard palate (Figure 1). The SARS-CoV-2 testing continued to remain positive in the emergency department. The patient was admitted to a specialized COVID-19 unit one month ago and was discharged home in an improved clinical condition after receiving high-dose corticosteroid treatment. One week later, he presented with a presumed nasal furuncle to the general surgeons and received oral antibiotic treatment without improvement. A computed tomography (CT) scan of the head was requested and the patient was referred for an ENT opinion. Nasal endoscopy showed dark-colored nasal crusts on the middle turbinate and the nasal septum. Radiologically, total opacification of all paranasal sinuses with erosions of the nasal septum and the hard palate and osteolytic regions of the right maxillary sinus was noted (Figure 2). The patient underwent limited surgical debridement and biopsy which confirmed the presence of invasive fungal rhinosinusitis (Figure 3). Magnetic resonance imaging of the head showed ischemic areas in the brainstem attributed to septic emboli (Figure 4). Postoperatively, he was treated with intravenous fluids, broad-spectrum antibiotics, and insulin therapy per local protocol. Intravenous liposomal amphotericin B was administered at a dosage of 3 mg/kg. Subcutaneous tinzaparin was added to the treatment plan after neurology consultation. Two extensive nasal debridement operations were performed under general anesthesia. Tissue specimens were sent for histological examination and culture, and the results confirmed the presence of *Staphylococcus aureus *and *Aspergillus niger*. The patient improved and was discharged on oral antifungals (posaconazole) and a fitted palatal prosthesis to prevent nasal regurgitation six weeks after admission. He discontinued the antifungals and the low-molecular-weight heparin and suffered an ischemic stroke a month later. His nasal examination revealed a well-healed nasal cavity during the weekly control after hospitalization. He is alive three months after the discharge.

## Discussion

The clinical manifestation of IFRS is characterized by a black nasal cavity and palatine eschars due to the vascular invasion of fungus. A typical finding in the late diagnosis of this infection is rhinocerebral mycosis which shows signs of vascular thrombosis and, as a consequence, necrosis of the involved tissues. Nasal endoscopy, imaging, and biopsies are crucial for rapid diagnosis. Treatment is based on intravenous antifungal medication, amphotericin B in mucormycosis, and voriconazole for* Aspergillus* treatment [2]. Surgical debridement with endoscopic sinus surgery should be performed repeatedly to achieve healthy sinus tissue. The diagnosis of the disease in the early stages has the best outcomes after correct treatment and minimizes mortality and morbidity. IFRS in the setting of COVID-19 infection is infrequently reported. The exact pathophysiological mechanisms of this co-infection are unknown. It has been suggested that the incidence of IFRS is higher in post-COVID-19 patients than in non-COVID-19 patients [3,4], notably, in immunocompetent patients. Research on SARS-CoV and SARS-CoV-2 showed remarkable similarities in their mechanisms, both increasing the incidence of fungal invasion. Aspergillosis of sinuses is reported among recently recovered patients from COVID-19 (30.6%) [5]. Other histopathologically proven mycoses were mucor (77.8%) and rhizopus. During the two-year ongoing pandemic, targeted therapy for COVID-19 patients is still under investigation. Corticosteroids have been proven to reduce the mortality rates of these patients, predisposing them to invasive fungal infections. Hyperglycemia has been highlighted as a factor affecting the function of neutrophils and monocytes leading to immune dysfunction. In addition, steroids that are included in the COVID-19 treatment protocols have an immunosuppressive effect by exacerbating an already dysregulated glycemic control. The European Organization for Research and treatment of Cancer and the Mycoses Study Group Education and Research Consortium mention that the use of cortisone at a dose of >0.3 mg/kg for at least three weeks during the past 60 days is shown to be a risk factor for invasive fungal disease [6]. Finally, high iron concentrations due to renal insufficiency create a favorable environment for fungal growth.

## Conclusions

IFRS is a rare entity with a high mortality rate. The exact association of this infection with COVID-19 remains undetermined. Physicians should consider the possibility of IFRS in patients with COVID-19 and underlying medical conditions, especially when they present with nasal symptoms. Therefore, we should raise awareness of the “red-alert” symptoms of a possible co-infection from fungal diseases in patients who received an immunosuppressive treatment for a recent COVID-19 infection.

## References

[1] Craig JR (2019). Updates in management of acute invasive fungal rhinosinusitis. Curr Opin Otolaryngol Head Neck Surg.

[2] Bakhshaee M, Bojdi A, Allahyari A, Majidi MR, Tavakol S, Najafzadeh MJ, Asghari M (2016). Acute invasive fungal rhinosinusitis: our experience with 18 cases. Eur Arch Otorhinolaryngol.

[3] Ismaiel WF, Abdelazim MH, Eldsoky I, Ibrahim AA, Alsobky ME, Zafan E, Hasan A (2021). The impact of COVID-19 outbreak on the incidence of acute invasive fungal rhinosinusitis. Am J Otolaryngol.

[4] Ebeid K, Gamea M, Allam AA, Shehata E (2021). Impact of COVID-19 on acute invasive fungal rhinosinusitis: a comparative study. Egypt J Ear Nose Throat Allied Sci.

[5] El-Kholy NA, El-Fattah AM, Khafagy YW (2021). Invasive fungal sinusitis in post COVID-19 patients: a new clinical entity. Laryngoscope.

[6] Donnelly JP, Chen SC, Kauffman CA (2020). Revision and update of the consensus definitions of invasive fungal disease from the European Organization for Research and Treatment of Cancer and the Mycoses Study Group Education and Research Consortium. Clin Infect Dis.

